# Crystal structure of aquadioxido(2-{[(2-oxido­ethyl)­imino]­meth­yl}phenol­ato-κ^3^ *O*,*N*,*O*′)molybdenum(VI)

**DOI:** 10.1107/S2056989015001231

**Published:** 2015-01-24

**Authors:** Sowmianarayanan Parimala, Parasuraman Selvam

**Affiliations:** a National Centre for Catalysis Research and Department of Chemistry, Indian Institute of Technology-Madras, Chennai 600 036, India; b New Industry Creation Hatchery Center, Tohoku University, Sendai 980 8579, Japan; c School of Science and Health, University of Western Australia, Sydney, Penrith, NSW 275, Australia

**Keywords:** crystal structure, dioxidomolybdenum(VI) complex, hydrogen bonding

## Abstract

The mononuclear title complex, [Mo(C_9_H_9_NO_2_)O_2_(H_2_O)], contains an Mo(VI) atom in a distorted octa­hedral coordination sphere defined by an Mo=O and an Mo—(OH_2_) bond to the axial ligands and two Mo—O bonds to phenolate and alcoholate O atoms, another Mo=O bond and one Mo—N bond to the imino N atom in the equatorial plane. The five-membered metalla-ring shows an envelope conformation. In the crystal, individual mol­ecules are connected into a layered arrangement parallel to (100) by means of O—H⋯O hydrogen bonds involving the water mol­ecule as a donor group and the O atoms of neighbouring complexes as acceptor atoms. These inter­actions lead to the formation of a three-dimensional network.

## Related literature   

For dioxidomolybdenum complexes used as potential oxidation catalysts for the epoxidation of alkenes, see: Sakthivel *et al.* (2005 ▸); Masteri-Farahani *et al.* (2006 ▸). For chiral molybdenum complexes, see: Burke (2008 ▸); Kühn *et al.* (2005 ▸). These compounds are good catalysts for the oxidation of organic compounds, see: Rayati *et al.* (2012 ▸). For heterogenization of polymer-supported molybdenum complexes, see: Sherrington *et al.* (2000 ▸); Maurya (2012 ▸), and for molybdenum systems on silica supports, see: Tangestaninejad *et al.* (2008 ▸).
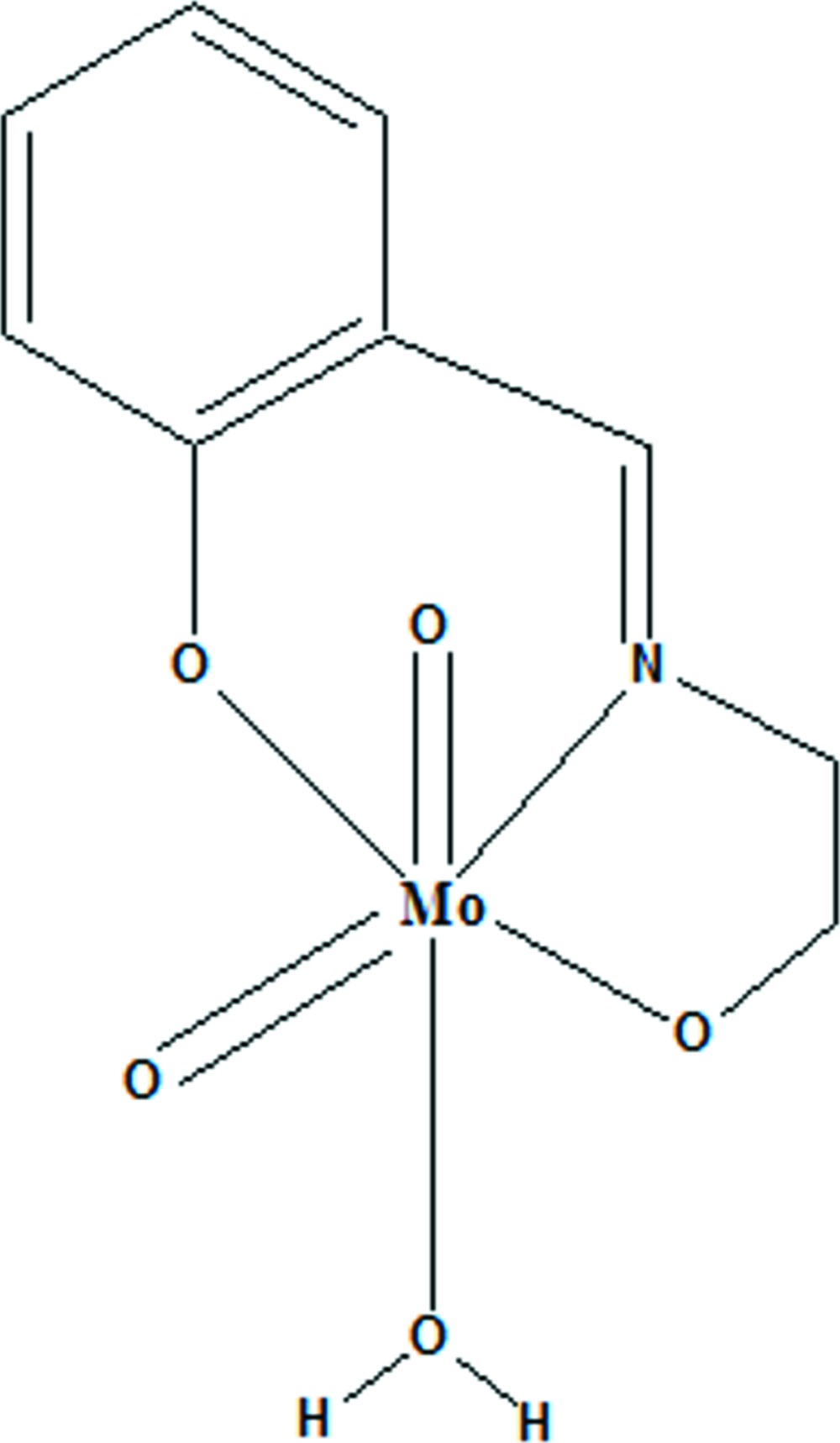


## Experimental   

### Crystal data  

[Mo(C_9_H_9_NO_2_)O_2_(H_2_O)]
*M* *_r_* = 309.13
Monoclinic, 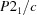

*a* = 14.9710 (3) Å
*b* = 6.7026 (1) Å
*c* = 10.8673 (2) Å
β = 99.486 (1)°
*V* = 1075.56 (3) Å^3^
*Z* = 4
Mo *K*α radiation
μ = 1.22 mm^−1^
*T* = 296 K
0.25 × 0.16 × 0.10 mm

### Data collection  

Bruker APEXII CCD diffractometer
Absorption correction: multi-scan (*SADABS*; Bruker, 2012 ▸) *T*_min_ = 0.813, *T*_max_ = 0.934
8837 measured reflections
2392 independent reflections
2263 reflections with *I* > 2σ(*I*)
*R*_int_ = 0.012

### Refinement  

*R*[*F* ^2^ > 2σ(*F* ^2^)] = 0.016
*wR*(*F* ^2^) = 0.042
*S* = 1.10
2392 reflections
153 parameters
H atoms treated by a mixture of independent and constrained refinement
Δρ_max_ = 0.31 e Å^−3^
Δρ_min_ = −0.30 e Å^−3^

### 

Data collection: *APEX2* (Bruker, 2012 ▸); cell refinement: *SAINT* (Bruker, 2012 ▸); data reduction: *SAINT*; program(s) used to solve structure: *SHELXS97* (Sheldrick, 2008 ▸); program(s) used to refine structure: *SHELXL97* (Sheldrick, 2015 ▸); molecular graphics: *ORTEP-3 for Windows* (Farrugia, 2012 ▸); software used to prepare material for publication: *publCIF* (Westrip, 2010 ▸).

## Supplementary Material

Crystal structure: contains datablock(s) I, New_Global_Publ_Block. DOI: 10.1107/S2056989015001231/wm5114sup1.cif

Structure factors: contains datablock(s) I. DOI: 10.1107/S2056989015001231/wm5114Isup2.hkl

**Fig. 1.**
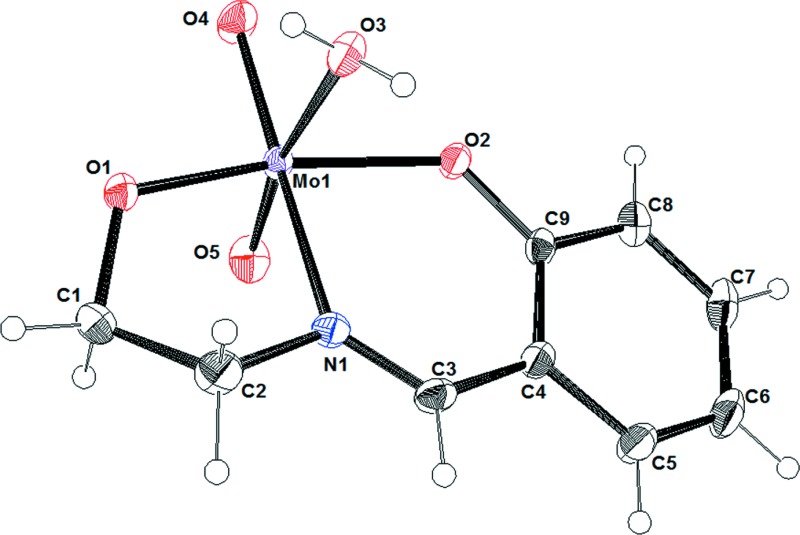
The molecular structure of the title compound with displacement ellipsoids drawn at the 30% probability level.

. DOI: 10.1107/S2056989015001231/wm5114fig1.tif
The mol­ecular structure of the title compound with displacement ellipsoids drawn at the 30% probability level.

**Fig. 2.**
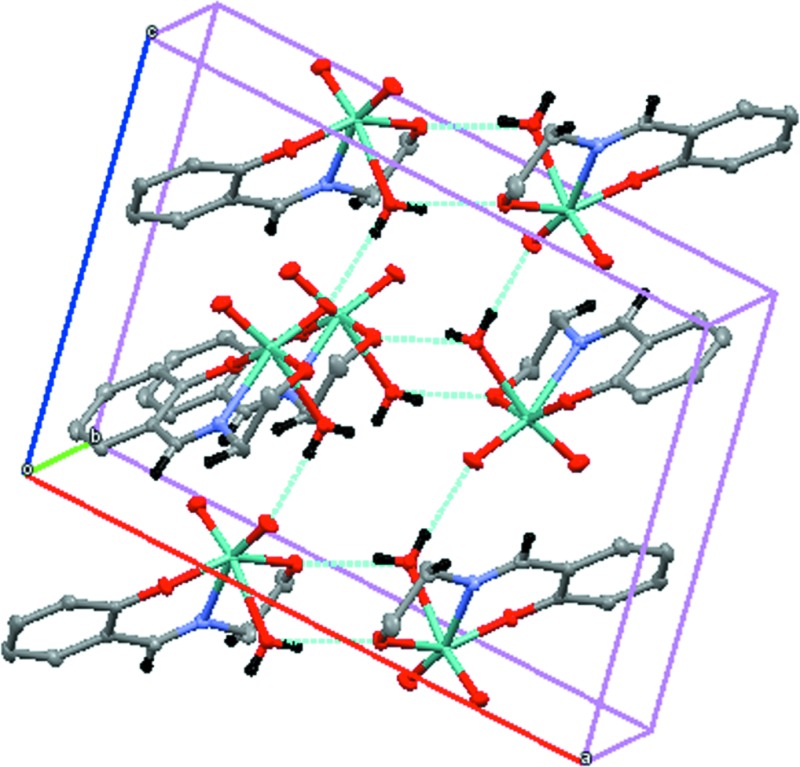
Unit-cell packing diagram of the title compound with hydrogen bonds shown as dashed lines. Hydrogen atoms not involved in hydrogen bonding are omitted for clarity.

. DOI: 10.1107/S2056989015001231/wm5114fig2.tif
Unit-cell packing diagram of the title compound with hydrogen bonds shown as dashed lines. Hydrogen atoms not involved in hydrogen bonding are omitted for clarity.

CCDC reference: 1044382

Additional supporting information:  crystallographic information; 3D view; checkCIF report

## Figures and Tables

**Table 1:** Selected bond lengths ()

Mo1O5	1.6902(14)
Mo1O4	1.7160(13)
Mo1O1	1.9438(12)
Mo1O2	1.9446(12)
Mo1N1	2.2652(14)
Mo1O3	2.3259(14)

**Table 2:** Hydrogen-bond geometry (, )

*D*H*A*	*D*H	H*A*	*D* *A*	*D*H*A*
O3H1*O*O1^i^	0.73(2)	1.97(3)	2.6656(19)	161(3)
O3H2*O*O4^ii^	0.78(3)	2.07(3)	2.8425(19)	173(3)

Symmetry codes: (i) 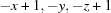; (ii) 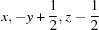.

## supplementary crystallographic information

### S1. Experimental

Molybdenyl acetylacetone (MoO_2_(acac)_2_) (4.03 g, 0.012 mol) dissolved in methanol (20 ml) was added to a refluxing solution of salicylaldehyde (2.62 ml, 0.012 mol) and ethanolamine (1.5 ml, 0.012 mol) in ethanol (30 ml). The mixture was refluxed for five hours, and the solvent removed under vacuum at room temperature. The resulting yellow solution was filtered, evaporated slowly, to yield yellow crystals. The crystals were purified by washing with ethanol/methanol mixture and dried at room temperature. The obtained crystals have incorporated water. The used solvents ethanol and methanol have not been dried prior to the reaction and thus contain water. Another source of water is the condensation reaction between salicylaldehyde and ethanolamine.

### S2. Refinement

All H atoms were identified from difference electron density maps. However, C-bound H atoms were treated as riding with C—H = 0.97 Å for (CH_2_) and C—H = 0.93 Å for aromatic H atoms, both with *U*_iso_(H) = 1.2*U*_eq_. The H atoms of the water molecule were refined freely.

### Crystal data

**Table:**

[Mo(C_9_H_9_NO_2_)O_2_(H_2_O)]	*F*(000) = 616
*M**_r_* = 309.13	*D*_x_ = 1.909 Mg m^−^^3^
Monoclinic, *P*2_1_/*c*	Mo *K*α radiation, λ = 0.71073 Å
*a* = 14.9710 (3) Å	Cell parameters from 6907 reflections
*b* = 6.7026 (1) Å	θ = 2.8–27.2°
*c* = 10.8673 (2) Å	µ = 1.22 mm^−^^1^
β = 99.486 (1)°	*T* = 296 K
*V* = 1075.56 (3) Å^3^	Block, yellow
*Z* = 4	0.25 × 0.16 × 0.10 mm

### Data collection

**Table:**

Bruker APEXII CCD diffractometer	2263 reflections with *I* > 2σ(*I*)
φ and ω scans	*R*_int_ = 0.012
Absorption correction: multi-scan (*SADABS*; Bruker, 2012)	θ_max_ = 27.2°, θ_min_ = 1.4°
*T*_min_ = 0.813, *T*_max_ = 0.934	*h* = −19→18
8837 measured reflections	*k* = −8→8
2392 independent reflections	*l* = −13→13

### Refinement

**Table:**

Refinement on *F*^2^	0 restraints
Least-squares matrix: full	Hydrogen site location: mixed
*R*[*F*^2^ > 2σ(*F*^2^)] = 0.016	H atoms treated by a mixture of independent and constrained refinement
*wR*(*F*^2^) = 0.042	*w* = 1/[σ^2^(*F*_o_^2^) + (0.0163*P*)^2^ + 0.8254*P*] where *P* = (*F*_o_^2^ + 2*F*_c_^2^)/3
*S* = 1.10	(Δ/σ)_max_ = 0.001
2392 reflections	Δρ_max_ = 0.31 e Å^−^^3^
153 parameters	Δρ_min_ = −0.30 e Å^−^^3^

### Special details

**Table:**

Geometry. All e.s.d.'s (except the e.s.d. in the dihedral angle between two l.s. planes) are estimated using the full covariance matrix. The cell e.s.d.'s are taken into account individually in the estimation of e.s.d.'s in distances, angles and torsion angles; correlations between e.s.d.'s in cell parameters are only used when they are defined by crystal symmetry. An approximate (isotropic) treatment of cell e.s.d.'s is used for estimating e.s.d.'s involving l.s. planes.

### Fractional atomic coordinates and isotropic or equivalent isotropic displacement parameters (Å^2^)

**Table:**

	*x*	*y*	*z*	*U*_iso_*/*U*_eq_	
C1	0.37800 (15)	−0.3126 (3)	0.4461 (2)	0.0394 (5)	
H1A	0.3281	−0.3758	0.4778	0.047*	
H1B	0.4281	−0.4060	0.4541	0.047*	
C2	0.34936 (14)	−0.2553 (3)	0.31132 (19)	0.0384 (5)	
H2A	0.4018	−0.2223	0.2734	0.046*	
H2B	0.3169	−0.3641	0.2649	0.046*	
C3	0.22629 (12)	−0.0467 (3)	0.22157 (16)	0.0288 (4)	
H3	0.2174	−0.1379	0.1562	0.035*	
C4	0.16677 (11)	0.1238 (3)	0.21392 (16)	0.0267 (4)	
C5	0.09354 (13)	0.1343 (3)	0.11473 (18)	0.0371 (4)	
H5	0.0846	0.0313	0.0567	0.044*	
C6	0.03507 (13)	0.2930 (4)	0.10178 (19)	0.0427 (5)	
H6	−0.0136	0.2961	0.0365	0.051*	
C7	0.04887 (13)	0.4486 (4)	0.1865 (2)	0.0404 (5)	
H7	0.0090	0.5561	0.1780	0.049*	
C8	0.12118 (13)	0.4458 (3)	0.28334 (18)	0.0331 (4)	
H8	0.1304	0.5525	0.3387	0.040*	
C9	0.18051 (11)	0.2837 (3)	0.29870 (15)	0.0244 (3)	
Mo1	0.32157 (2)	0.08935 (2)	0.49415 (2)	0.02134 (5)	
N1	0.29025 (10)	−0.0807 (2)	0.31202 (14)	0.0254 (3)	
O1	0.40476 (8)	−0.13539 (19)	0.51476 (12)	0.0286 (3)	
O2	0.25056 (8)	0.29088 (18)	0.39282 (11)	0.0275 (3)	
O3	0.42590 (10)	0.2153 (2)	0.37755 (13)	0.0306 (3)	
O4	0.37757 (10)	0.2275 (2)	0.61586 (12)	0.0372 (3)	
O5	0.23417 (9)	−0.0248 (2)	0.54594 (13)	0.0392 (3)	
H1O	0.4715 (17)	0.176 (4)	0.395 (2)	0.037 (7)*	
H2O	0.4144 (17)	0.221 (4)	0.305 (3)	0.048 (7)*	

### Atomic displacement parameters (Å^2^)

**Table:**

	*U*^11^	*U*^22^	*U*^33^	*U*^12^	*U*^13^	*U*^23^
C1	0.0454 (11)	0.0219 (9)	0.0463 (12)	0.0080 (8)	−0.0058 (9)	−0.0057 (8)
C2	0.0417 (11)	0.0331 (10)	0.0382 (11)	0.0118 (8)	−0.0003 (8)	−0.0154 (8)
C3	0.0325 (9)	0.0312 (9)	0.0219 (8)	−0.0039 (7)	0.0024 (7)	−0.0059 (7)
C4	0.0239 (8)	0.0331 (9)	0.0223 (8)	−0.0025 (7)	0.0019 (6)	0.0033 (7)
C5	0.0344 (10)	0.0460 (12)	0.0274 (9)	−0.0061 (9)	−0.0045 (8)	0.0021 (9)
C6	0.0301 (10)	0.0601 (14)	0.0341 (10)	−0.0005 (9)	−0.0064 (8)	0.0154 (10)
C7	0.0325 (10)	0.0508 (13)	0.0384 (11)	0.0142 (9)	0.0068 (8)	0.0183 (10)
C8	0.0351 (10)	0.0363 (10)	0.0286 (9)	0.0097 (8)	0.0075 (7)	0.0061 (8)
C9	0.0231 (8)	0.0295 (9)	0.0207 (8)	0.0010 (7)	0.0038 (6)	0.0059 (7)
Mo1	0.02586 (8)	0.02184 (8)	0.01569 (8)	0.00375 (5)	0.00161 (5)	−0.00128 (5)
N1	0.0283 (7)	0.0234 (7)	0.0241 (7)	0.0011 (6)	0.0031 (6)	−0.0047 (6)
O1	0.0297 (6)	0.0241 (6)	0.0295 (6)	0.0054 (5)	−0.0023 (5)	−0.0007 (5)
O2	0.0306 (6)	0.0243 (6)	0.0247 (6)	0.0054 (5)	−0.0035 (5)	−0.0023 (5)
O3	0.0250 (7)	0.0404 (8)	0.0251 (7)	0.0017 (6)	0.0007 (5)	0.0052 (6)
O4	0.0479 (8)	0.0381 (8)	0.0219 (6)	0.0062 (6)	−0.0049 (6)	−0.0085 (6)
O5	0.0370 (7)	0.0451 (8)	0.0382 (8)	0.0037 (6)	0.0144 (6)	0.0072 (7)

### Geometric parameters (Å, º)

**Table:**

C1—O1	1.425 (2)	C6—H6	0.9300
C1—C2	1.507 (3)	C7—C8	1.380 (3)
C1—H1A	0.9700	C7—H7	0.9300
C1—H1B	0.9700	C8—C9	1.396 (2)
C2—N1	1.468 (2)	C8—H8	0.9300
C2—H2A	0.9700	C9—O2	1.3397 (19)
C2—H2B	0.9700	Mo1—O5	1.6902 (14)
C3—N1	1.275 (2)	Mo1—O4	1.7160 (13)
C3—C4	1.443 (3)	Mo1—O1	1.9438 (12)
C3—H3	0.9300	Mo1—O2	1.9446 (12)
C4—C9	1.406 (3)	Mo1—N1	2.2652 (14)
C4—C5	1.407 (2)	Mo1—O3	2.3259 (14)
C5—C6	1.370 (3)	O3—H1O	0.73 (2)
C5—H5	0.9300	O3—H2O	0.78 (3)
C6—C7	1.384 (3)		
			
O1—C1—C2	107.86 (16)	C7—C8—H8	119.8
O1—C1—H1A	110.1	C9—C8—H8	119.8
C2—C1—H1A	110.1	O2—C9—C8	117.79 (16)
O1—C1—H1B	110.1	O2—C9—C4	122.62 (15)
C2—C1—H1B	110.1	C8—C9—C4	119.56 (16)
H1A—C1—H1B	108.4	O5—Mo1—O4	107.11 (7)
N1—C2—C1	105.88 (15)	O5—Mo1—O1	97.32 (6)
N1—C2—H2A	110.6	O4—Mo1—O1	96.15 (6)
C1—C2—H2A	110.6	O5—Mo1—O2	97.01 (6)
N1—C2—H2B	110.6	O4—Mo1—O2	102.33 (6)
C1—C2—H2B	110.6	O1—Mo1—O2	152.09 (5)
H2A—C2—H2B	108.7	O5—Mo1—N1	90.18 (6)
N1—C3—C4	124.35 (16)	O4—Mo1—N1	161.73 (6)
N1—C3—H3	117.8	O1—Mo1—N1	75.35 (5)
C4—C3—H3	117.8	O2—Mo1—N1	80.78 (5)
C9—C4—C5	118.37 (17)	O5—Mo1—O3	166.38 (6)
C9—C4—C3	122.93 (15)	O4—Mo1—O3	86.41 (6)
C5—C4—C3	118.66 (17)	O1—Mo1—O3	82.47 (5)
C6—C5—C4	121.4 (2)	O2—Mo1—O3	78.05 (5)
C6—C5—H5	119.3	N1—Mo1—O3	76.56 (5)
C4—C5—H5	119.3	C3—N1—C2	121.04 (15)
C5—C6—C7	119.60 (18)	C3—N1—Mo1	127.21 (12)
C5—C6—H6	120.2	C2—N1—Mo1	111.56 (11)
C7—C6—H6	120.2	C1—O1—Mo1	117.80 (11)
C8—C7—C6	120.62 (19)	C9—O2—Mo1	133.92 (11)
C8—C7—H7	119.7	Mo1—O3—H1O	114.5 (19)
C6—C7—H7	119.7	Mo1—O3—H2O	120.8 (18)
C7—C8—C9	120.37 (19)	H1O—O3—H2O	109 (3)
			
O1—C1—C2—N1	−46.2 (2)	C3—C4—C9—O2	0.6 (3)
N1—C3—C4—C9	8.2 (3)	C5—C4—C9—C8	0.8 (3)
N1—C3—C4—C5	−174.14 (18)	C3—C4—C9—C8	178.44 (16)
C9—C4—C5—C6	−1.7 (3)	C4—C3—N1—C2	−178.44 (18)
C3—C4—C5—C6	−179.50 (19)	C4—C3—N1—Mo1	7.0 (3)
C4—C5—C6—C7	1.2 (3)	C1—C2—N1—C3	−149.68 (18)
C5—C6—C7—C8	0.3 (3)	C1—C2—N1—Mo1	25.66 (19)
C6—C7—C8—C9	−1.3 (3)	C2—C1—O1—Mo1	51.2 (2)
C7—C8—C9—O2	178.67 (17)	C8—C9—O2—Mo1	152.01 (14)
C7—C8—C9—C4	0.7 (3)	C4—C9—O2—Mo1	−30.1 (2)
C5—C4—C9—O2	−177.10 (16)		

### Hydrogen-bond geometry (Å, º)

**Table:**

*D*—H···*A*	*D*—H	H···*A*	*D*···*A*	*D*—H···*A*
O3—H1*O*···O1^i^	0.73 (2)	1.97 (3)	2.6656 (19)	161 (3)
O3—H2*O*···O4^ii^	0.78 (3)	2.07 (3)	2.8425 (19)	173 (3)

Symmetry codes: (i) −*x*+1, −*y*, −*z*+1; (ii) *x*, −*y*+1/2, *z*−1/2.
