# Supplementary figures and images for: Crystal structure of aquadioxido(2-{[(2-oxido­ethyl)­imino]­meth­yl}phenol­ato-κ3 O,N,O′)molybdenum(VI)
Source: Acta Crystallogr E Crystallogr Commun. 2015 Jan 24;71(Pt 2):m35–6. doi: 10.1107/S2056989015001231 (PMC4384576; doi:10.1107/S2056989015001231)

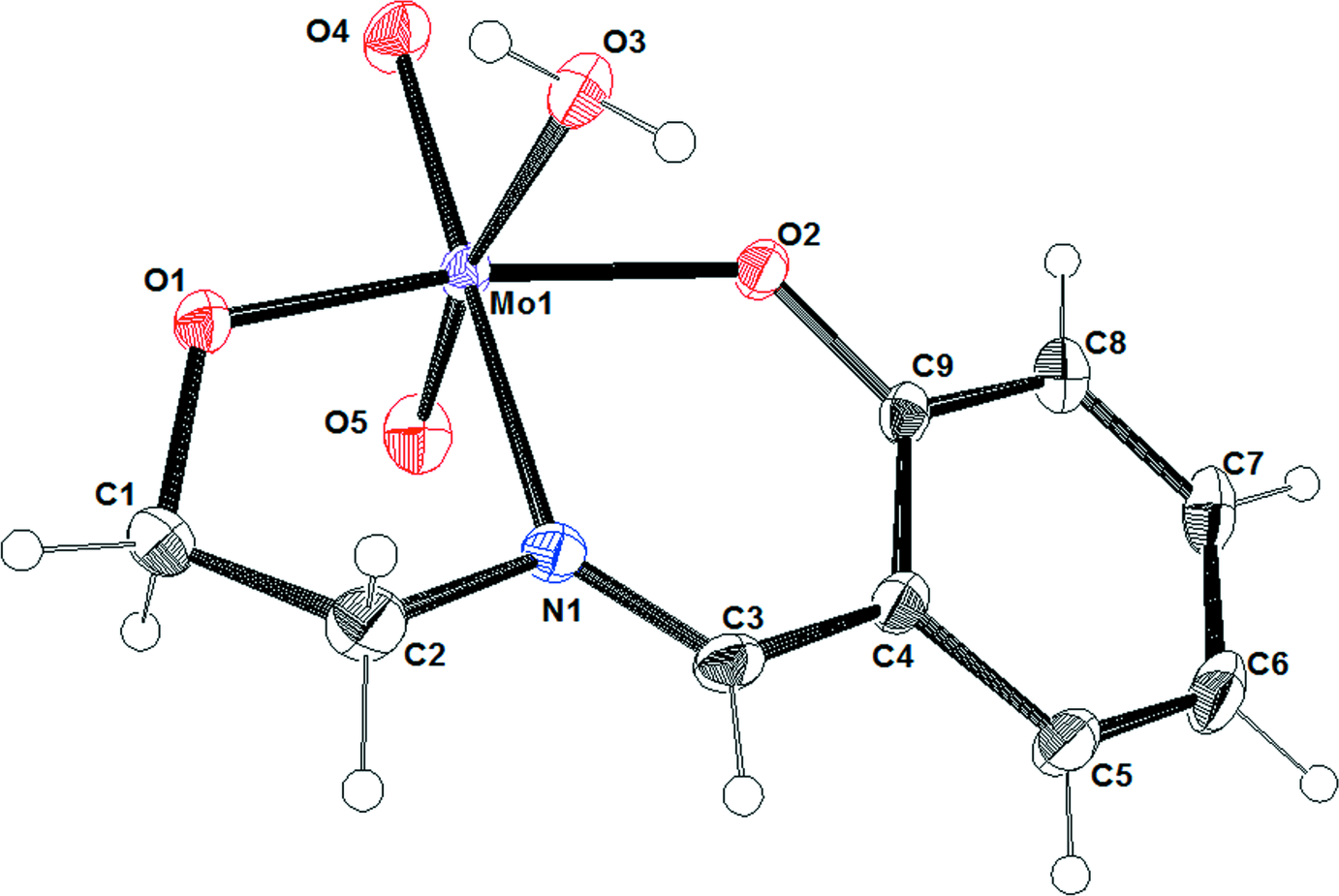

Supplement: Supplementary file 3 [file e-71-00m35-fig1.tif]

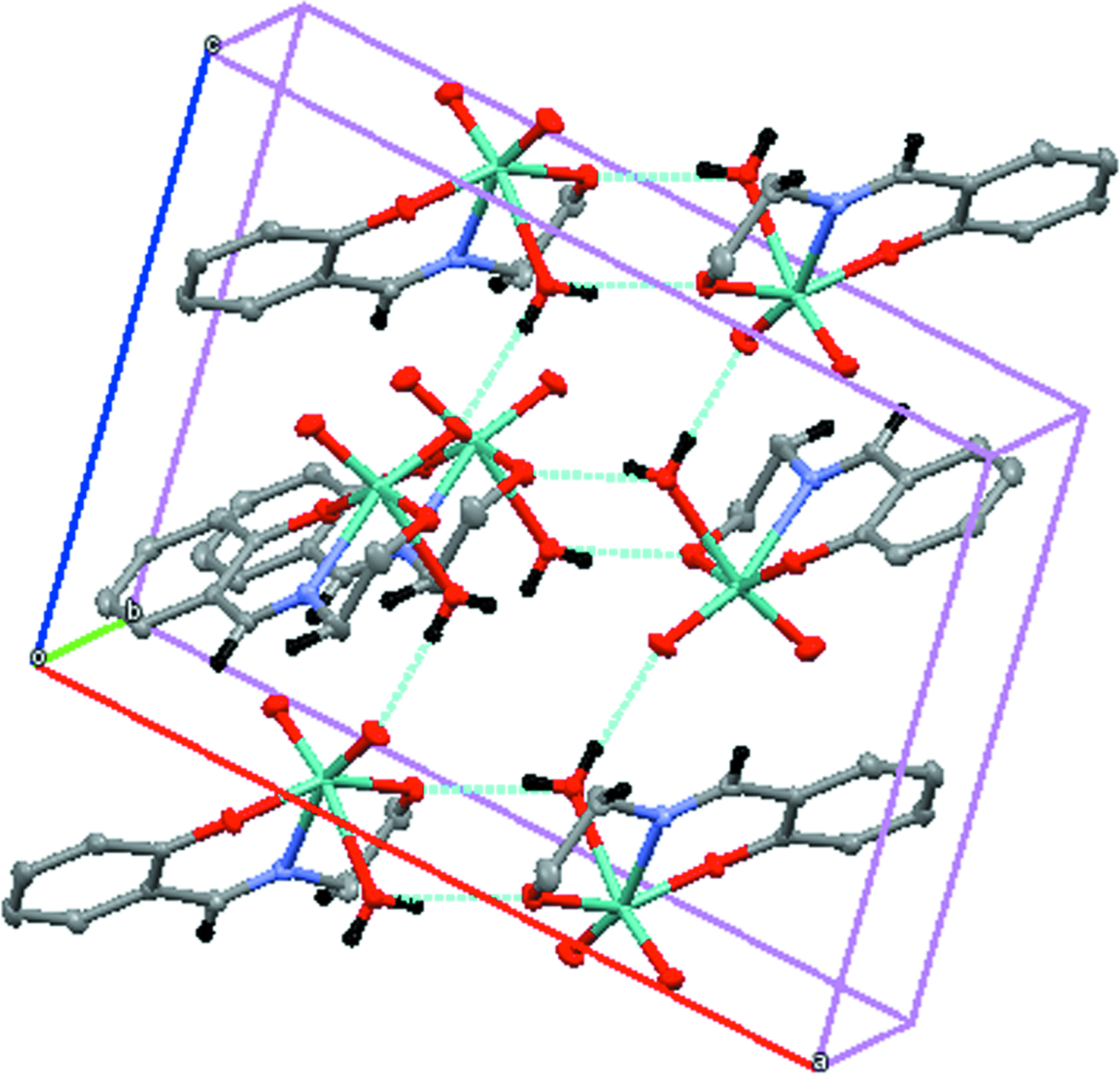

Supplement: Supplementary file 4 [file e-71-00m35-fig2.tif]
